# Thermodynamics and polarity-driven properties of fluorinated cyclopropanes

**DOI:** 10.3762/bjoc.21.137

**Published:** 2025-08-29

**Authors:** Matheus P Freitas

**Affiliations:** 1 Department of Chemistry, Institute of Natural Sciences, Federal University of Lavras, 37200-900, Lavras, MG, Brazil

**Keywords:** cyclopropane, fluorination, polarity, theoretical calculations

## Abstract

Cyclopropane is a significant alicyclic motif, widely utilized in medicinal chemistry, while fluorination serves as a powerful tool to modulate properties that enhance the performance of pharmaceuticals and materials. This quantum-chemical study explores the energetic implications of fluorinating cyclopropane, providing insights into molecular characteristics arising from the polar C–F bond. Isodesmic reactions revealed that the conversion of cyclopropane and methyl fluoride into mono-, di-, tri-, tetra-, penta-, and hexafluorinated cyclopropanes is exothermic, except for the all-*cis*-1,2,3-trifluorocyclopropane (**1.2.3-c.c.**). Compounds featuring geminal fluorines are particularly stabilized due to anomeric-like *n*_F_ → σ*_CF_ interactions. Generally, *cis*-C–F bonds are less favored than their *trans* counterparts, not primarily because of steric repulsion, but due to reduced stabilizing electron-delocalization interactions. Among the series, **1.2.3-c.c.** stands out as the most polar compound, enabling unique stacking interactions between its electrostatically complementary negative and positive faces. These interactions are mediated through electrostatic hydrogen bonds. This "Janus-like" polarity also facilitates interactions with ions, particularly sodium and chloride. These findings contribute valuable insights for the rational design of drugs and advanced materials, particularly those whose properties rely on the polarity and spatial arrangement of C–F bonds within a cyclopropane framework.

## Introduction

**Figure 1:**
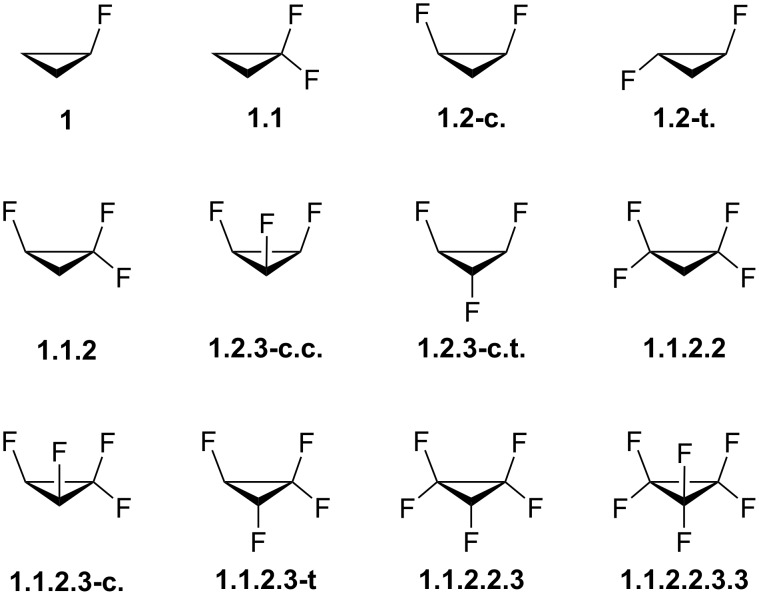
Structures of fluorinated cyclopropanes evaluated in this study through quantum chemical methods.

Cyclopropane, the smallest cycloalkane, has a rigid structure that prevents ring interconversion, unlike its larger analogs. It is widely used in pharmaceuticals and has attracted significant interest in medicinal chemistry, particularly when fluorinated [1–6]. The highly polar C–F bond in fluorinated cyclopropanes destabilizes the ring and alters its overall polarity, with these effects varying based on the amount, position, and orientation of the fluorine substituents [7–8]. Mondal and colleagues have extensively reviewed additional properties of fluorinated cyclopropanes, including the conformational behavior of substituents attached to a fluorinated cyclopropyl ring [9]. Building on these insights, the relative stability of all possible fluorinated cyclopropanes, from mono- to perfluorocyclopropane (Figure 1), was quantum chemically evaluated in this study. Polarity-dependent properties of the most polar compound were also analyzed to inform potential applications.

The all-*cis*-1,2,3-trifluorocyclopropane (**1.2.3-c.c.**) is expected to be the most polar compound within the series of fluorinated cyclopropanes, as the C–F bonds are aligned in the same direction. Similarly, all-*cis*-1,2,3,4,5,6-hexafluorocyclohexane is both the most polar and the least stable isomer among the 1,2,3,4,5,6-hexafluorocyclohexanes [10]. Although it was initially synthesized via a multistep reaction, it is now readily obtainable through the catalytic hydrogenation of hexafluorobenzene [11]. Its Janus-face-like structure has demonstrated unprecedented potential, particularly due to its ability to form ion complexes and self-assemble into a stacking arrangement [12–14].

Therefore, understanding the energetics and polarity of fluorinated cyclopropanes is crucial for elucidating the intramolecular forces that govern the stability of molecular structures. Moreover, these insights can guide the prediction of molecular properties, paving the way for the design of innovative applications. Fluorinated cyclopropanes, with their unique electronic and structural characteristics, hold significant potential in the development of new drugs, advanced materials like liquid crystals, and efficient ion carriers. These applications highlight their versatility and underscore the importance of further research into their behavior and properties.

## Results and Discussion

An isodesmic reaction is defined as a reaction in which the total number of each type of bond remains identical in the reactants and products [15]. While such reactions are theoretically possible, they are not always experimentally feasible. Their primary purpose is to provide insight into the favorability of specific processes. In this study, we employ the following isodesmic reaction to assess whether the fluorination of cyclopropane is a stabilizing or destabilizing process:

cyclopropane + *n* CH_3_F → *n*-fluorocyclopropane + *n* CH_4_

A prior quantum-chemical investigation of the isodesmic fluorination reaction involving cyclopropane and 2,2-difluoropropane to form 1,1-difluorocyclopropane and propane demonstrated that the substitution is endothermic and, therefore, destabilizing [8]. This destabilization was attributed to the greater *s*-character of the orbital involved in bonding to the substituent. In contrast, our calculations at the B3LYP-GD3BJ/6-311++G(d,p) level revealed that the reactions leading to the fluorinated cyclopropanes depicted in Figure 1 are predominantly exothermic and spontaneous, with the exception of the reaction forming compound **1.2.3-c.c.** (Table 1).

**Table 1:** Standard Gibbs free energies (Δ*G*^0^) and enthalpy changes (Δ*H*^0^) for the isodesmic reactions, relative Gibbs free (*G*^0^_rel_) and electronic energies (*E*_rel_, comparing the isomers of di-, tri-, and tetrafluorinated cyclopropanes depicted in Figure 1), as well as Lewis (*E*_L_) and non-Lewis (*E*_NL_) contributions to the total electronic energy. Molecular dipole moments (μ) are also included. Energies are reported in kcal mol^−1^, and dipole moments are given in debye (D).

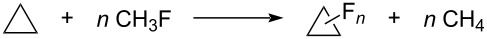							

Compound	Δ*G*^0^	Δ*H*^0^	*G* ^0^_rel_	*E*_rel_	*E*_L_	*E*_NL_	μ

**1**	−4.7	−4.8					1.98
**1.1**	−13.7	−14.0	0.0	0.0	30.6	−30.6	2.54
**1.2-c.**	−4.5	−4.8	9.2	9.2	9.2	0.0	3.15
**1.2-t.**	−7.5	−7.7	6.2	6.3	8.3	−2.0	1.33
**1.1.2**	−12.9	−13.3	0.0	0.0	34.2	−34.2	2.53
**1.2.3-c.c.**	0.7	0.0	13.5	13.4	13.4	0.0	4.17
**1.2.3-c.t.**	−5.1	−5.5	7.8	7.9	14.2	−6.4	1.39
**1.1.2.2**	−17.8	−18.5	0.0	0.0	32.0	−32.0	2.37
**1.1.2.3-c.**	−7.2	−8.0	10.7	10.6	10.6	0.0	2.88
**1.1.2.3-t.**	−9.9	−10.6	7.9	7.9	13.1	−5.2	1.00
**1.1.2.2.3**	−11.7	−12.7					1.62
**1.1.2.2.3.3**	−13.2	−14.6					0.00

The most favored reactions are those involving geminal fluorines, particularly in the formation of compound **1.1.2.2**. Geminal fluorination is highly stabilizing due to the presence of two anomeric-like interactions, *n*_F_ → σ*_CF_ [16]. According to a natural bond orbital (NBO) analysis, this electron delocalization accounts for a stabilization energy of 14.3 kcal mol^−1^ per interaction in compound **1.1**. Similar stabilization values are observed in other compounds containing geminal fluorines. Additionally, fluorination increases the C–(F)–C bond angle compared to the C–C–C angle in cyclopropane (60°). Specifically, the bond angles widen to 61.5° in compound **1** and 63.5° in compound **1.1**, moving closer to the ideal tetrahedral angle.

Despite maximizing the number of geminal fluorines, compound **1.1.2.2.3.3** (the perfluorinated cyclopropane) is not the most readily formed, as its reaction is less exothermic compared to the formation of compounds **1.1.2.2** and **1.1**. This behavior can be attributed to the all-*cis* arrangement of the C–F bonds on both faces of the ring, which intensifies steric and dipolar repulsions. The unfavorable nature of this arrangement is further highlighted by the thermodynamic parameters (Δ*H*^0^ and Δ*G*^0^) for the reaction leading to compound **1.2.3-c.c.**, which indicate its formation is energetically unfavorable. However, this does not preclude its synthesis, as the all-*cis*-1,2,3-trifluorocyclopropane framework has been successfully prepared in earlier studies [17].

To better understand the intramolecular interactions that govern the stability of fluorinated cyclopropanes, the sets of di-, tri-, and tetrafluorinated cyclopropane isomers will be analyzed by decomposing the electronic energy (*E*_rel_) into Lewis (*E*_L_) and non-Lewis (*E*_NL_) components. Notably, *E*_rel_ closely correlates with the relative standard Gibbs free energies (*G*^0^). The *E*_L_ term represents classical steric and electrostatic contributions, while the *E*_NL_ term accounts for electron delocalization effects. Among the difluorocyclopropanes, the stability order is **1.1** > **1.2-t.** > **1.2-c.**, highlighting the destabilizing influence of 1,2-*syn*-diaxial repulsion in the **1.2-c.** isomer. A similar interaction is observed in *cis*-1,3-difluorocyclobutane, where the diaxial conformer is destabilized, and only the equatorial form is present at equilibrium [18]. While **1.1** exhibits the largest *E*_L_ term due to steric and electrostatic factors, it is also significantly stabilized by electron delocalization, as previously noted. Conversely, **1.2-c.** experiences greater destabilization from steric and dipolar repulsion compared to **1.2-t.** and receives less stabilization from electron delocalization. The electron delocalization interactions in these systems are primarily *syn*- and *antiperiplanar* σ_CH_ → σ*_CF_ interactions. Although the latter is more stabilizing and more prevalent in **1.2-c**, the overall *E*_NL_ contribution favors **1.2-t** over **1.2-c**.

For trifluorocyclopropanes, the stability order is **1.1.2** > **1.2.3-c.t.** > **1.2.3-c.c.** As previously discussed, **1.1.2** benefits significantly from anomeric-like interactions, which greatly enhance the *E*_NL_ term. Interestingly, while the most polar compound, **1.2.3-c.c.**, is less stable than **1.2.3-c.t.**, this is not due to a higher *E*_L_ term but rather to less effective stabilization from electron delocalization interactions. This finding is somewhat surprising, as one might expect **1.2.3-c.c.** to exhibit greater steric and dipolar repulsion involving the polar C–F bonds. Finally, a similar pattern is observed for the tetrafluorinated cyclopropanes. Compound **1.1.2.2**, which maximizes anomeric-like interactions, is the most stable isomer. Meanwhile, **1.1.2.3-t.** is more stable than **1.1.2.3-c.**, not due to weaker steric or dipolar repulsion, but because of more favorable electron delocalization interactions.

**Figure 2:**
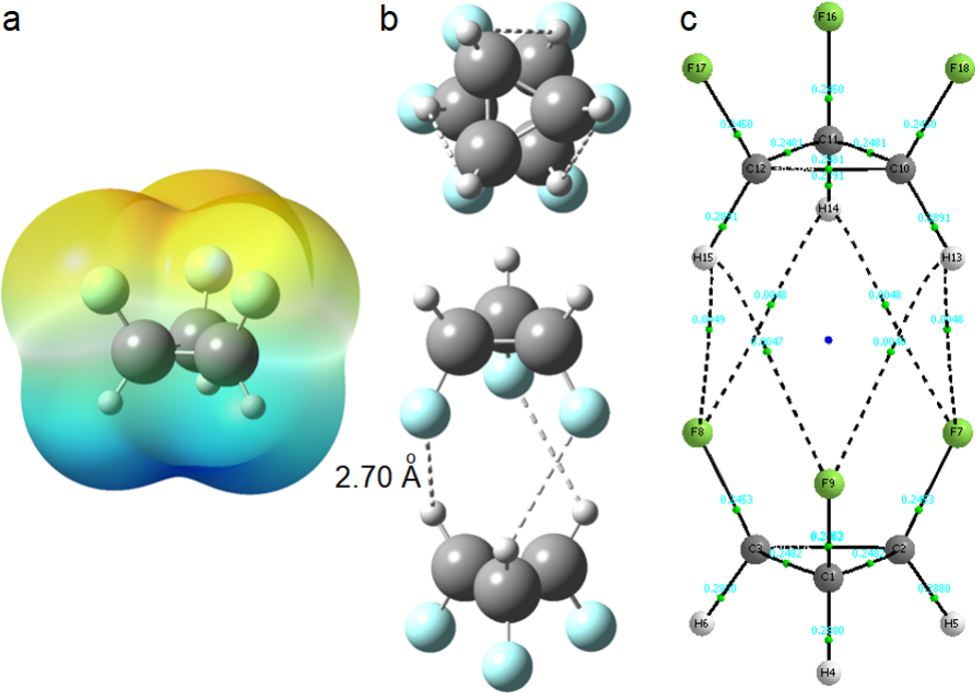
(a) Electrostatic potential map of **1.2.3-c.c.**, highlighting the negative region (top side) and positive region (bottom side). (b) Top and side views of two **1.2.3-c.c.** molecules interacting, with an H···F distance of 2.70 Å. (c) QTAIM plot illustrating bond paths between hydrogen and fluorine atoms, indicative of electrostatic hydrogen bonds.

Among the compounds shown in Figure 1, **1.2.3-c.c.** is the most polar, with a calculated dipole moment of 4.17 D. Polarity is a key molecular property, as it influences solubility, lipophilicity, and various material characteristics. For example, liquid crystals used in LCD devices rely on specific properties such as dielectric anisotropy, where the molecular dipole moment aligns parallel to the molecule’s long axis [19]. Compound **1.2.3-c.c.** exhibits a "Janus"-face-like structure, characterized by a positively charged region on one side and a negatively charged region on the other, as illustrated by the electrostatic potential in Figure 2. This unique structural feature facilitates molecular stacking, wherein the negative region of one molecule interacts with the positive region of another. Dimerization of **1.2.3-c.c.** is thermodynamically (Δ*H*^0^) favored – the dimer is 3.8 kcal mol^−1^ more stable than two isolated molecules – and an atoms in molecules (AIM) analysis reveals a bond path between hydrogen and fluorine atoms, with an electron density of 0.0048 at the bond critical point (Figure 2). Based on AIM charges (*q*_H_ = +0.0851 and *q*_F_ = −0.6003) and a H···F distance of 2.70 Å, this interaction is consistent with electrostatic hydrogen bonding. Consequently, similar to the behavior observed in the crystalline form of all-*cis*-1,2,3,4,5,6-hexafluorocyclohexane [10], **1.2.3-c.c.** is expected to self-assemble in the condensed phase.

**Figure 3:**
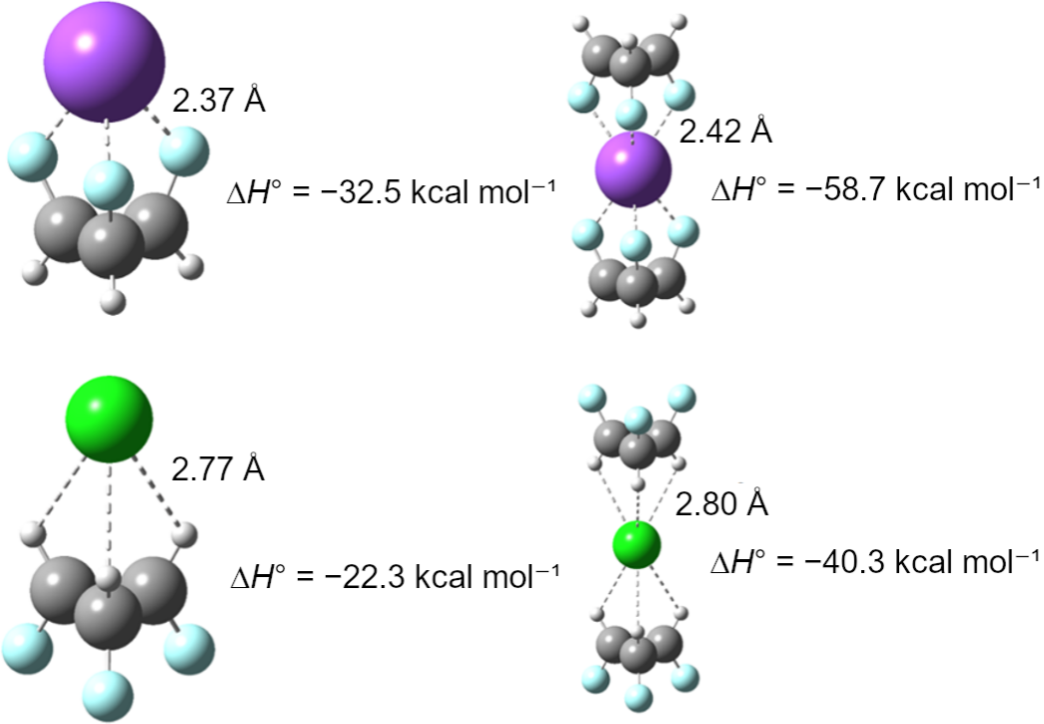
Calculated complexes of **1.2.3-c.c.** with Na^+^ (top) and Cl^−^ (bottom).

Given the "Janus"-face-like structure of **1.2.3-c.c.**, it is capable of interacting with ions and potentially acting as both an anion and a cation carrier. To evaluate this property, Na^+^ and Cl^−^ ions were simulated in interaction with one and two molecules of **1.2.3-c.c.**, forming complexes akin to a "sandwich," as illustrated in Figure 3. In these complexes, the cation coordinates with the fluorine atoms, while the anion interacts with the positively charged hydrogen atoms. The complexation is thermodynamically favorable when compared to the isolated species (ions and **1.2.3-c.c.**), as indicated by the Δ*H*^0^ of formation: −32.5 kcal mol^−1^ for **1.2.3-c.c.**–Na^+^ (1:1), −58.7 kcal mol^−1^ for **1.2.3-c.c.**–Na^+^–**1.2.3-c.c.** (2:1), −22.3 kcal mol^−1^ for **1.2.3-c.c.**–Cl^−^ (1:1), and −40.3 kcal mol^−1^ for **1.2.3-c.c.**–Cl^−^–**1.2.3-c.c.** (2:1). Based on these energies, atomic charges, and the observed interaction distances, the coordination of fluorines with Na^+^ appears to be stronger than the interaction of hydrogens with Cl^−^. These findings suggest that, similar to all-*cis*-1,2,3,4,5,6-hexafluorocyclohexane [13], the **1.2.3-c.c.** motif has significant potential to transport ions across specific media due to its distinctly polarized faces.

## Conclusion

This study highlights the significant influence of fluorination on the stability, polarity, and electronic properties of cyclopropane, offering valuable insights through detailed quantum-chemical analyses. The isodesmic reaction approach demonstrated that fluorination is predominantly exothermic, with geminal fluorines providing the greatest stability due to strong anomeric-like interactions that enhance electron delocalization. Notably, the all-*cis*-1,2,3-trifluorocyclopropane (**1.2.3-c.c.**) was identified as the most polar compound, with its Janus-like electrostatic potential enabling unique intermolecular interactions, such as stacking, electrostatic hydrogen bonding, and complexation with ions. The interplay of steric effects, dipolar repulsion, and electron delocalization emerged as critical factors in governing the stability of isomeric fluorinated cyclopropanes. These findings not only deepen the understanding of intramolecular forces in fluorinated systems but also provide a molecular framework for the design of advanced applications. Fluorinated cyclopropanes exhibit considerable potential in pharmaceuticals, where polarity influences drug solubility and activity, and in material sciences, particularly for the development of liquid crystals, ion carriers, and other technologies dependent on precise molecular properties.

## Computational Details

The fluorinated cyclopropanes shown in Figure 1 were optimized using density functional theory (DFT) at the B3LYP-GD3BJ/6-311++G(d,p) level [20–22], which includes dispersion corrections to improve the modeling of nonbonding interactions [23]. The same computational protocol was applied to cyclopropane, methane, and methyl fluoride, used as reference molecules in the isodesmic reactions. This methodology has proven reliable for predicting the energetics and properties of organofluorine compounds [24]. Frequency calculations confirmed the absence of imaginary frequencies, ensuring the structures correspond to true minima, and provided standard Gibbs free energies. For the dimer of **1.2.3-c.c.** and its complexes with Na^+^ and Cl^−^, basis set superposition error was addressed using counterpoise corrections. Natural bond orbital (NBO) analysis was conducted using the NBO 7.0 program [25] to identify donor–acceptor interactions responsible for electron delocalization within the systems. The NOSTAR/NBODEL keyword was employed to calculate deletion energies by excluding unoccupied orbitals (antibonding and Rydberg), offering insights into the contributions of non-Lewis electron delocalization and its interplay with Lewis-type bonding in the total electronic energy. All calculations were performed using the Gaussian 16 software suite [26]. Quantum theory of atoms in molecules (QTAIM) analyses were carried out with the AIMAll program [27].

## Supporting Information

File 1 Standard orientations and Gibbs free energies for the studied compounds.

## Data Availability

All data that supports the findings of this study is available in the published article and/or the supporting information of this article.
