# Supplementary material for: Thermodynamics and polarity-driven properties of fluorinated cyclopropanes
Source: Beilstein J Org Chem. 2025 Aug 29;21:1742–7. doi: 10.3762/bjoc.21.137 (PMC12415895; doi:10.3762/bjoc.21.137)
Supplement: File 1 — Standard orientations and Gibbs free energies for the studied compounds. [file Beilstein_J_Org_Chem-21-1742-s001.pdf]

BEILSTEIN JOURNAL OF ORGANIC CHEMISTRY

Supporting Information

for

Thermodynamics and polarity-driven properties of fluorinated cyclopropanes

Matheus P. Freitas

Beilstein J. Org. Chem. **2025,** *21,* 1742–1747. doi:10.3762/bjoc.21.137

Standard orientations and Gibbs free energies for the studied compounds

License and Terms: This is a supporting information file under the terms of the Creative Commons Attribution License (https://creativecommons.org/licenses/by/4.0). Please note that the reuse, redistribution and reproduction in particular requires that the author(s) and source are credited and that individual graphics may be subject to special legal provisions.
The license is subject to the *Beilstein Journal of Organic Chemistry* terms and conditions: (https://www.beilstein-journals.org/bjoc/terms)

Table of contents

Pages S2–S12. Standard Cartesian coordinates for the optimized geometries of the studied compounds and their corresponding standard Gibbs free energies ($\Delta G°$), calculated at the B3LYP-GD3BJ/6-311++G(d,p) level of theory.

Methane

0 1

C 0.00000000 0.00000000 0.00000000

H 0.62966400 0.62966400 0.62966400

H -0.62966400 -0.62966400 0.62966400

H -0.62966400 0.62966400 -0.62966400

H 0.62966400 -0.62966400 -0.62966400

G^0 = -40.508639 hartrees

Fluoromethane

0 1

C 0.00000000 0.00000000 -0.63952800

H 0.00000000 1.03485900 -0.98850500

H -0.89621400 -0.51743000 -0.98850500

H 0.89621400 -0.51743000 -0.98850500

F 0.00000000 0.00000000 0.75585400

G^0 = -139.775939 hartrees

Cyclopropane

0 1

C 0.75411800 0.43539000 0.00000000

C 0.00000000 -0.87076200 0.00000000

C -0.75411000 0.43538200 0.00000000

H 1.26312600 0.72925200 0.90980600

H 1.26312600 0.72925200 -0.90980600

H -0.00002000 -1.45850500 0.90980400

H -0.00002000 -1.45850500 -0.90980400

H -1.26313200 0.72922100 -0.90980300

H -1.26313200 0.72922100 0.90980300

G^0 = -117.882098 hartrees

Fluorocyclopropane (**1**)

0 1

C 0.24692800 0.00000000 0.48053200

C -0.87739100 -0.76228800 -0.13149200

C -0.87739000 0.76228800 -0.13149300

H 0.40406100 0.00000000 1.55230000

H -1.55828000 -1.29308600 0.52275800

H -0.67332300 -1.24793100 -1.07756100

H -0.67332100 1.24793100 -1.07756100

H -1.55827800 1.29308800 0.52275700

F 1.45625200 0.00000000 -0.19422000

G^0 = -217.156923 hartrees

1,1-Difluorocyclopropane (**1.1**)

0 1

C 0.00000300 0.17413500 0.00000000

C 0.77587800 -1.07856600 0.00000000

C -0.77588700 -1.07855600 0.00000000

H 1.27525500 -1.35971000 0.91828700

H 1.27525500 -1.35971000 -0.91828700

H -1.27526800 -1.35969300 -0.91828700

H -1.27526800 -1.35969300 0.91828700

F 0.00000300 0.96315100 -1.10650200

F 0.00000300 0.96315100 1.10650200

G^0 = -316.438503 hartrees

cis-1,2-Difluorocyclopropane (**1.2cis**)

0 1

C -0.74846400 0.25019400 0.42642800

C -0.00007800 1.19722800 -0.47032600

C 0.74847100 0.25024400 0.42635200

H -0.00006200 2.25840000 -0.25267700

H -0.00017100 0.92914400 -1.52048900

H 1.29450800 0.60533100 1.29255800

F -1.38142500 -0.81024900 -0.17258600

F 1.38148900 -0.81019000 -0.17260000

H -1.29443000 0.60509000 1.29256100

G^0 = -316.423836 hartrees

trans-1,2-Difluorocyclopropane (**1.2trans**)

0 1

C 0.59911800 -0.09939500 -0.43630100

C -0.00000100 1.20962200 0.00000000

C -0.59911800 -0.09939500 0.43630200

H -0.51790200 1.78398100 -0.75847200

H 0.51789800 1.78398300 0.75847200

F 1.79933000 -0.48955100 0.11824100

H 0.50313900 -0.41051800 -1.46924300

H -0.50313700 -0.41051700 1.46924300

F -1.79932900 -0.48955200 -0.11824100

G^0 = -316.428603 hartrees

1,1,2-Trifluorocyclopropane (**1.1.2**)

0 1

C 0.53173000 0.02593400 0.03698700

C -0.18607400 -0.88368100 0.96767600

C -0.79910900 -0.48320500 -0.37536200

H -0.61782700 -0.41459800 1.84403600

H 0.16170600 -1.90273600 1.08350500

F 1.63176300 -0.41724200 -0.61970700

H -0.86427900 -1.21343300 -1.17334700

F -1.86083100 0.37863400 -0.35804600

F 0.67808000 1.32488300 0.36330900

G^0 = -415.704493 hartrees

All-*cis*-1,2,3-trifluorocyclopropane (**1.2.3ciscis**)

0 1

C 0.48174600 0.72777400 0.59736800

C -0.87128800 0.05340700 0.59725000

C 0.38928800 -0.78110300 0.59739100

H -1.50253100 0.09210400 1.47819500

H 0.67136500 -1.34735100 1.47828700

F 0.70788500 -1.41963200 -0.56241700

F 0.87591000 1.32260400 -0.56245400

F -1.58359300 0.09696400 -0.56255000

H 0.83086300 1.25535800 1.47825200

G^0 = -415.682905 hartrees

cis,*trans*,*trans*-1,2,3-Trifluorocyclopropane (**1.2.3cistrans**)

0 1

C 0.21119500 0.75722000 0.44411500

C -0.76528100 0.00001000 -0.39558600

C 0.21117900 -0.75721500 0.44411900

H -0.12334300 -1.25907800 1.34501900

F 1.20898900 -1.42848200 -0.20575600

F 1.20902700 1.42845500 -0.20575800

H -0.12331800 1.25909700 1.34501100

H -0.59336900 0.00000800 -1.46643600

F -2.09607500 0.00001400 -0.05287300

G^0 = -415.692053 hartrees

1,1,2,2-Tetrafluorocyclopropane (**1.1.2.2**)

0 1

C 0.03579800 0.74370000 0.00000000

C 1.34914300 0.00000200 0.00000000

C 0.03580000 -0.74370000 0.00000000

F -0.34311600 -1.40934600 1.10649500

F -0.34311600 1.40934500 1.10649600

H 1.91386200 0.00000400 0.92500600

F -0.34311600 1.40934500 -1.10649600

F -0.34311600 -1.40934600 -1.10649500

H 1.91386200 0.00000400 -0.92500600

G^0 = -514.979693 hartrees

cis-1,1,2,3-Tetrafluorocyclopropane (**1.1.2.3cis**)

0 1

C -0.69861800 0.00000000 0.02762700

C 0.40351700 -0.77000400 -0.62913100

C 0.40351600 0.77000300 -0.62913200

F -1.92691800 -0.00000100 -0.54083800

H 0.21411500 -1.28396800 -1.56538900

F -0.78125300 0.00000100 1.36271500

F 1.29415600 1.40720100 0.17320500

H 0.21411400 1.28396600 -1.56539100

F 1.29415700 -1.40720000 0.17320700

G^0 = -514.962693 hartrees

trans-1,1,2,3-Tetrafluorocyclopropane (**1.1.2.3trans**)

0 1

C 0.00000200 0.63889700 0.00000000

C -0.53444500 -0.65315000 0.53837800

C 0.53444000 -0.65315400 -0.53837700

F -0.75251600 1.39810900 -0.81538600

F 0.75252400 1.39810600 0.81538600

F 1.78994200 -1.07390700 -0.21391500

H 0.22640200 -0.91562700 -1.54548400

F -1.78994700 -1.07389800 0.21391400

H -0.22640600 -0.91562600 1.54548500

G^0 = -514.967122 hartrees

Pentafluorocyclopropane (**1.1.2.2.3**)

0 1

C -0.75152400 -0.26979000 0.07083900

C 0.75153300 -0.26977100 0.07083700

C -0.00000900 0.65316300 -0.87621900

F -1.42573700 -1.30776600 -0.45158800

F -1.38803000 0.25536900 1.12197000

F -0.00003100 1.98628400 -0.63678000

H -0.00000900 0.38432900 -1.92854100

F 1.42577000 -1.30773100 -0.45159100

F 1.38802800 0.25540600 1.12196600

G^0 = -614.237180 hartrees

Hexafluorocyclopropane (**1.1.2.2.3.3**)

0 1

C 0.44053600 -0.76302800 0.00000000

C -0.88110900 -0.00000700 0.00000000

C 0.44049500 0.76305900 0.00000000

F 0.81335100 -1.40868700 1.10782300

F 0.81335100 -1.40868700 -1.10782300

F 0.81335100 1.40870000 -1.10781100

F -1.62667700 -0.00002200 1.10781600

F -1.62667700 -0.00002200 -1.10781600

F 0.81335100 1.40870000 1.10781100

G^0 = -713.506980 hartrees

Bis-all-*cis*-1,2,3-trifluorocyclopropane

0 1 0 1 0 1

C(Fragment=2) -2.74761000 -0.85790700 -0.14971100

C(Fragment=2) -2.74663400 0.56099700 -0.66758100

C(Fragment=2) -2.74512200 0.30006800 0.82023100

H(Fragment=2) -3.62079700 -1.49025500 -0.25978300

H(Fragment=2) -3.61884800 0.97499800 -1.15977900

H(Fragment=2) -3.61609700 0.52172700 1.42577000

F(Fragment=2) -1.57461800 1.00927500 -1.20554600

F(Fragment=2) -1.57175400 0.53861500 1.47619600

F(Fragment=2) -1.57659700 -1.54892600 -0.27216800

C(Fragment=1) 1.54142000 -0.30317700 -0.81705000

C(Fragment=1) 1.54415400 0.86043300 0.14561100

C(Fragment=1) 1.54193800 -0.55511300 0.67197800

H(Fragment=1) 0.65810800 -0.52044900 -1.40446500

H(Fragment=1) 0.66299100 1.48085000 0.25079800

H(Fragment=1) 0.65908400 -0.95351400 1.15600200

F(Fragment=1) 2.70867300 1.56378300 0.26420800

F(Fragment=1) 2.70468800 -1.01318400 1.22252800

F(Fragment=1) 2.70368400 -0.55458200 -1.48848500

G^0 = -831.359838 hartrees

1.2.3ciscis–Na^+

1 1 0 1 1 1

C(Fragment=1) -1.08821500 0.87050800 -0.00468900

C(Fragment=1) -1.08658400 -0.43013200 0.75882000

C(Fragment=1) -1.08953500 -0.44100600 -0.74945700

H(Fragment=1) -1.92232200 1.55926600 -0.00774900

H(Fragment=1) -1.91851500 -0.77051600 1.36058400

H(Fragment=1) -1.92378800 -0.79023800 -1.34288600

F(Fragment=1) 0.13807300 1.51590200 -0.01182200

F(Fragment=1) 0.13640800 -0.76736100 -1.30721300

F(Fragment=1) 0.14167300 -0.74841500 1.31650800

Na(Fragment=2) 1.96411300 0.00037600 -0.00138700

G^0 = -577.823964 hartrees

1.2.3ciscis–Cl^-

-1 1 0 1 -1 1

C(Fragment=1) 0.39521700 -0.58492500 -0.64525300

C(Fragment=1) 0.39640800 -0.26734600 0.82956400

C(Fragment=1) 0.39430700 0.85105600 -0.18290200

H(Fragment=1) -0.49798300 -0.99559800 -1.09750800

H(Fragment=1) -0.49556100 -0.45551600 1.41262900

H(Fragment=1) -0.49983200 1.44703800 -0.31071600

F(Fragment=1) 1.57699800 -1.06384700 -1.17621500

F(Fragment=1) 1.57519300 1.55130500 -0.33449700

F(Fragment=1) 1.57938000 -0.48546900 1.50853900

Cl(Fragment=2) -2.83566800 -0.00038500 0.00039400

G^0 = -876.026513 hartrees

1.2.3ciscis–Na^+–**1.2.3ciscis**

1 1 0 1 0 1 1 1

C(Fragment=1) 3.11230800 0.34094400 -0.78304200

C(Fragment=1) 3.10770200 0.46719100 0.72000600

C(Fragment=1) 3.06587000 -0.89686100 0.07761200

H(Fragment=1) 3.95547500 0.82896000 1.28629700

H(Fragment=1) 3.88212400 -1.60414500 0.13950900

F(Fragment=1) 1.82627100 -1.50442900 0.12486000

F(Fragment=1) 1.90629800 0.65887800 -1.37816900

F(Fragment=1) 1.89824400 0.87928900 1.24637500

H(Fragment=1) 3.96370400 0.60356800 -1.39658400

C(Fragment=2) -3.10584800 0.45908500 0.72732800

C(Fragment=2) -3.06242400 -0.90020900 0.07496600

C(Fragment=2) -3.11729300 0.34358300 -0.77651100

H(Fragment=2) -3.95243500 0.81357500 1.29993300

H(Fragment=2) -3.87523900 -1.61145700 0.13605200

H(Fragment=2) -3.97329000 0.60745000 -1.38309000

F(Fragment=2) -1.89557300 0.87189700 1.25132300

F(Fragment=2) -1.91598400 0.66985300 -1.37587900

F(Fragment=2) -1.81995100 -1.50354900 0.11145600

Na(Fragment=3) 0.00036600 0.07565500 -0.01308800

G^0 = -993.531128 hartrees

1.2.3ciscis–Cl^-–**1.2.3ciscis**

-1 1 0 1 0 1 -1 1

C(Fragment=1) 3.24887600 -0.68483600 -0.53832100

C(Fragment=1) 3.26082800 -0.12303300 0.86194400

C(Fragment=1) 3.28124200 0.80847900 -0.32483000

H(Fragment=1) 2.36876600 -0.19067900 1.47028800

H(Fragment=1) 2.40368900 1.39827900 -0.55391000

F(Fragment=1) 4.47372300 1.44589900 -0.59066400

F(Fragment=1) 4.41430600 -1.27282300 -0.97955800

F(Fragment=1) 4.43645000 -0.24948100 1.56964800

H(Fragment=1) 2.34819000 -1.14858800 -0.91811300

C(Fragment=2) -3.26190000 -0.08935600 0.86628900

C(Fragment=2) -3.28138200 0.79555900 -0.35558600

C(Fragment=2) -3.24805600 -0.70478200 -0.51126100

H(Fragment=2) -2.37065700 -0.13269900 1.47806700

H(Fragment=2) -2.40331700 1.37565300 -0.60609500

H(Fragment=2) -2.34703300 -1.18270000 -0.87217000

F(Fragment=2) -4.43833800 -0.18967900 1.57676100

F(Fragment=2) -4.41308600 -1.30962400 -0.93046500

F(Fragment=2) -4.47334600 1.42219500 -0.64762300

Cl(Fragment=3) 0.00031300 0.07353900 0.00174300

G^0 = -1291.728312 hartrees

Na^+

G^0 = -162.101996 hartrees

Cl^-

G^0 = -460.318750 hartrees
